# Histotype-Dependent Oligodendroglial PrP Pathology in Sporadic CJD: A Frequent Feature of the M2C “Strain”

**DOI:** 10.3390/v13091796

**Published:** 2021-09-09

**Authors:** Ellen Gelpi, Sigrid Klotz, Nuria Vidal-Robau, Gerda Ricken, Günther Regelsberger, Thomas Ströbel, Ognian Kalev, Marlene Leoni, Herbert Budka, Gabor G. Kovacs

**Affiliations:** 1 Division of Neuropathology and Neurochemistry, Department of Neurology, Medical University of Vienna, 1090 Vienna, Austria; sigrid.klotz@meduniwien.ac.at (S.K.); gerda.ricken@meduniwien.ac.at (G.R.); guenther.regelsberger@meduniwien.ac.at (G.R.); thomas.stroebel@meduniwien.ac.at (T.S.); herbert.budka@meduniwien.ac.at (H.B.); 2 Medical University of Vienna, Austrian Reference Center for Human Prion Diseases (OERPE), 1090 Vienna, Austria; 3 Department of Neurology, Bellvitge University Hospital, L’Hospitalet de Llobregat, 08908 Barcelona, Spain; nuria.virob@gmail.com; 4 Department of Neuropathology/Pathology, Kepler Universitäts Klinikum, 4020 Linz, Austria; Ognian.Kalev@kepleruniklinikum.at; 5 Department of Neuropathology/Pathology, Medical University Graz, 8036 Graz, Austria; marlene.leoni@medunigraz.at; 6 Tanz Centre for Research in Neurodegenerative Disease, University of Toronto, Toronto, ON M5T 0S8, Canada; Gabor.Kovacs@uhnresearch.ca; 7 Department of Laboratory Medicine and Pathobiology and Department of Medicine, University of Toronto, Toronto, ON M5T 0S8, Canada; 8 Laboratory Medicine Program & Krembil Brain Institute, University Health Network, Toronto, ON M5T 1M8, Canada

**Keywords:** Creutzfeldt-Jakob disease, CJD, PrP, prion, histotype, glia, oligodendrocytes

## Abstract

In sporadic Creutzfeldt-Jakob disease, molecular subtypes are neuropathologically well identified by the lesioning profile and the immunohistochemical PrP^d^ deposition pattern in the grey matter (histotypes). While astrocytic PrP pathology has been reported in variant CJD and some less frequent histotypes (e.g., MV2K), oligodendroglial pathology has been rarely addressed. We assessed a series of sCJD cases with the aim to identify particular histotypes that could be more prone to harbor oligodendroglial PrP^d^. Particularly, the MM2C phenotype, in both its more “pure” and its mixed MM1+2C or MV2K+2C forms, showed more frequent oligodendroglial PrP pathology in the underlying white matter than the more common MM1/MV1 and VV2 histotypes, and was more abundant in patients with a longer disease duration. We concluded that the MM2C strain was particularly prone to accumulate PrP^d^ in white matter oligodendrocytes.

## 1. Introduction

In human prion diseases, attention has been mainly focused on neuronal dysfunction, as PrP^c^ is enriched in synapses and its pathology positively correlates with the neurodegenerative process and clinical symptoms [1]. Astroglial pathology is an important feature of variant CJD and pathological astroglial PrP deposits represent, in addition to florid plaques, a key histopathological hallmark [2]. Moreover, the deposition of disease-associated PrP (PrP^d^) has been described in astrocytes [3], particularly in MV2K cases [4]. In contrast, oligodendrocytic PrP pathology has rarely been described. Fernandez-Vega et al. [5] reported the presence of nuclear and perinuclear PrP^d^ in oligodendrocytes in the frontal white matter in a 66-year-old man with an otherwise classical VV2-histotype, who had a disease duration of 4,5 months. We also observed the presence of oligodendroglial PrP^d^ pathology in the white matter of some sporadic CJD cases. This finding prompted us to analyze in more detail whether some specific CJD histotypes might be more prone to accumulate PrP^d^ in this particular glial cell type.

## 2. Materials and Methods

We screened the different basic histotypes of sporadic CJD: 20 MM/MV1, 10 VV2, 15 mixed MM/MV1 + 2C, 10 mixed MV2K + 2C and 10 MM2C. Demographical details of the patients and disease duration are given in Table 1. We assessed formalin-fixed, formic-acid-decontaminated, and paraffin-embedded tissue sections from the frontal and occipital lobe and paid particular attention to white matter pathology. Immunohistochemistry was performed by applying the anti-PrP 12F10 antibody (1:1000, epitope aa 142–160, CEA, Gif-sur-Yvette Cedex, France), after appropriate tissue pretreatment. This included a three-tiered tissue pretreatment based on 10 min hydrated autoclaving at 121 °C, 5 min, 96% formic acid, and 5 min proteinase K (5 μg/mL in TRIS) at 4 °C, prior to anti-PrP antibody incubation. In parallel we evaluated the PrP^d^ deposition pattern in the overlying cortex as synaptic, perineuronal, patchy perivacuolar, plaque-like and/or Kuru-type plaques, and assessed its intensity in a semiquantitative scale as follows: 0 = absent, 1 = mild, 2 = moderate, 3 = extensive. In selected cases that harbored oligodendroglial PrP^d^ pathology, we extended the anti-PrP antibody panel and included the monoclonal antibody 3F4 (1:500, epitope 109–112, Senetek PLC, CA, USA), 6H4 (1:500, epitope 144–152, Prionics, Schlieren ZH, Switzerland), KG9 (1:1000, epitope 140–180, TSE Resource Centre, Edinburgh, UK), and L42 (1:300, epitope 141–159 IgG1 FRC for Virus Diseases of Animals, sheep recPrP; Dr. M.H. Groschup, Tübingen, Germany). The Dako Envision Kit (DAKO, Glostrup, Denmark) was used as secondary system and diaminobenzidine as chromogen. Double immunofluorescence was performed on selected cases combining anti-PrP 12F10 and the oligodendroglia marker anti-TPPP/p25 (anti-tubulin polymerization promoting protein TPPP/p25, a protein that was expressed mainly in differentiated oligodendrocytes of the CNS [6], non-commercial antibody, rabbit, 1:250) as well as anti-PrP 12F10 and anti-GFAP antibodies (rabbit, 1:1500, DAKO), applying Alexa Fluor 488 goat-anti-mouse antibody (1:800, Jackson Immunoresearch, PA, USA) and Cy3 goat-anti-rabbit (1:1000, Jackson Immunoresearch, PA, USA) as secondary antibodies, in addition to DAPI nuclear stain (1 μg/mL, Thermo Fisher Scientific, MA, USA). Incubation of the antibodies was performed overnight at 4 °C. Autofluorescence was blocked with 1% aqueous sodium borohydride solution (4 min) and 1% sudan black B solution (5 min).

## 3. Results

**Figure 1:**
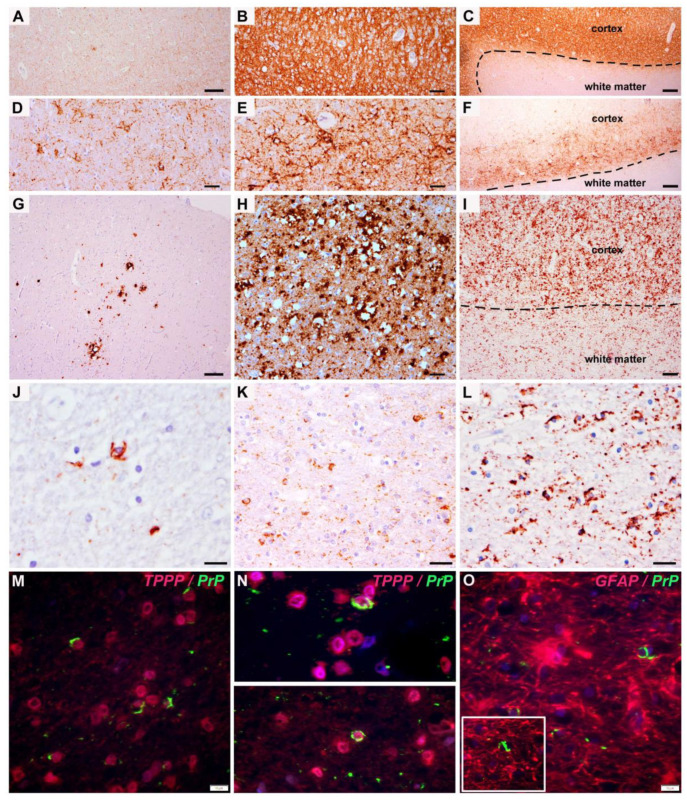
PrPd immunoreactivity patterns. A-C (MM1/MV1): Diffuse synaptic pattern, characteristic of MM/MV1 histotype ranging from mild (**A**) to extensive (**B**) deposits. (**C**) A relatively distinct border between cortical grey matter (upper part) and white matter (lower part) is observed in this subtype. No oligodendroglial pathology is usually detected. (**D**–**F**) (VV2/MV2): Perineuronal pattern, characteristic of VV2 (and MV2K) histotype in its milder (**D**) and more intense (**E**) form. This pattern has a typical deep laminar distribution (**F**). Here also, the border between cortical grey and white matter is relatively sharp, although some neuronal processes extend into the immediately underlying white matter. G-I (MM2C): Patchy perivacuolar pattern, characteristic of MM2C histotype, can appear as a focal feature (**G**), usually in mixed MM1+2C forms) or be widespread in bona fide MM2C (**H**). In some cases with very extensive cortical pathology, there might be a blurring of the grey/white matter boundary (**I**) and pathological PrP deposits can be abundant in the underlying white matter. (**J**–**L**) At higher magnification, PrP deposits can be identified in single (**J**) or multiple (**K**,**L**) white matter glial cells with oligodendroglial morphology. These aggregates show ring-like, coiled-body-like or more amorphous morphologies. (**M**–**O**) Double immunofluorescence: semilunar or ring-like glial PrP^d^ deposits (green signal) were clearly within TPPP/p25 positive oligodendrocyte (M, N: red signal; Patient 7/MV2K+2C from Table 1) but not within GFAP positive astrocytes (O, red signal); true co-localization of PrP^d^, however, occurred rarely with TPPP/p25 (lower panel in N). *Scale bars: A, G: 50 μm; B, D, E, H: 20 μm; C, F, I: 100 μm; J: 15 μm; K, L, M, N, O: 10 μm.*

We identified PrP^d^ pathology in white matter glial cells with oligodendroglia morphology only in cases with the MM2C (+1) (90% MM2C, 27% MM1 + 2C) and the mixed MV2K+C histotype (70%). The oligodendroglial pathology was particularly evident in cases with extensive patchy-perivacuolar PrP deposits in the overlying cortex (Figure 1H,I). The deposits were primarily cytoplasmatic and ring- or comma-shaped (Figure 1J–L), and were associated with fine-punctuate PrP^d^ deposits in the white matter reminiscent of axonal deposits. Moreover, oligodendroglial PrP^d^ deposits were comparable with coiled bodies observed in the four-repeat tauopathies, such as progressive supranuclear palsy, corticobasal degeneration or argyrophilic grain disease, or even glial cytoplasmic alpha-synuclein inclusions of MSA, as some PrP^d^ aggregates appeared coarser or microglobular. We did not observe an obvious nuclear PrP^d^ immunoreactivity. In contrast to tau and alpha-synuclein inclusions in oligodendroglia, white matter oligodendroglial PrP^d^ pathology was not visible in immunostaining for p62/ubiquitin and these deposits were not argyrophilic.

A comparable immunoreactivity pattern was identified when applying different anti-PrP antibodies directed to different epitopes such as KG9, 6H4 and L42, while they were not well identified with the 3F4 antibody, which had a poorer performance globally. In mixed MM/MV1+2C cases, only when a high amount of patchy-perivacuolar deposits were present did single oligodendrocytes harbor ring-shaped and granular cytoplasmic PrP^d^ immunoreactivity. In contrast, cases with only focal confluent vacuoles and focal patchy PrP^d^ deposits had no obvious oligodendroglial PrP inclusions (Table 1).

Oligodendroglial PrP^d^ accumulation was observed only in the subcortical white matter along axonal profiles, but was not visible in perivascular oligodendrocytes or perineuronal satellite oligodendrocytes within the cortex. Double immunofluorescence combining anti-TPPP/p25 and PrP (12F10) antibodies supported the oligodendroglial nature of cells harboring PrP^d^ aggregates (Figure 1M,N). In contrast, GFAP+ astrocytes did not show PrP^d^ accumulation within their cytoplasm (Figure 1O). The presence of oligodendroglial pathology was not related to age or sex, but was more frequently observed in cases with a longer disease duration. We could not identify oligodendroglial inclusions in MM/MV1, VV2 or pure MV2K cases. In MM/MV1 cases with extensive diffuse synaptic PrP deposits and prominent spongiform change, neuronal loss and gliosis, some ramified microglial cells at the cortico-subcortical boundary contained granular cytoplasmic PrP, but were not seen beyond that boundary.

## 4. Discussion

Our findings demonstrate that particular CJD histotypes, mainly those dominated by PrP^d^ type 2 with abundant patchy-perivacuolar deposits in the cortex (M2C “strain”), may be prone to accumulate PrP^d^ in oligodendroglial cells, particularly in patients with a long disease duration, and support earlier evidence of oligodendroglial involvement in some CJD cases. The presence of oligodendroglial PrP^d^ was not related to the cortical area analysed (frontal or occipital) but to the PrP^d^ deposition pattern and its intensity in the overlying cortex.

MM2C patients typically manifest disease at older ages, present with progressive dementia, and have longer disease durations than classical MM1 or VV2 patients [7,8]. The total amounts of 14-3-3 and tau proteins are usually increased in CSF, but the RT-QuIC assay for PrP may result negative [9]. Patients are therefore frequently misdiagnosed with Alzheimer’s disease or vascular/mixed dementia [8]. Neuropathological studies reveal prominent cortical pathology with large confluent vacuoles and patchy-perivacuolar PrP^d^ deposits with a relative sparing of the brainstem and cerebellum. Cortical MRI hyperintensities correlate well with this anatomical distribution. About one third of MM/MV1 patients also show focal areas of confluent vacuoles with a patchy-perivacuolar PrP deposition pattern [10], but are clinically indistinguishable from more “pure” MM1 cases, except maybe for the presence of more pronounced cortical hyperintensities in MRI in the areas with mixed pathology. MM2C features can also accompany some MV2K cases and here again, cortical hyperintensities may correlate to the foci of large confluent vacuoles [11,12].

By PrP^d^ immunohistochemistry, the cortical–subcortical boundary appears relatively sharply demarcated in typical MM/MV1 (Figure 1C), while in extensive MM2C patterns it appears blurred and PrP^d^ deposits frequently extend into the white matter (Figure 1I). The deep laminar perineuronal pattern in VV2 may also project the delicate neuronal processes into the underlying white matter (Figure 1F) or show plaque-like or coarse deposits along the axons, but PrP^d^ does not accumulate in oligodendroglial cytoplasm. At the cortico–subcortical boundary of some MM1/MV1 cases with extensive PrP^d^ pathology, microglia may harbor PrP deposits, as they do in the cortex [3,13]. However, in more distant white matter, we observed non-argyrophilic and p62/ubiquitin negative, morphologically “coil-like” bodies in oligodendrocytes only in M2C cases.

Glial cells play an important role in neuronal homeostasis, connectivity and plasticity [14,15,16]. Oligodendrocytes, besides insulating and supporting axons through the myelin sheath are also important regulators of signal transmission and synaptic function. Oligodendrocytes also interact with GABAergic interneurons of the cortex, which represent almost 50% of myelin content in the upper cortical layers [17,18,19], and are frequently affected in the early disease stages of CJD [20,21,22,23]. As both oligodendrocytes and axons need their mutual input for proper functioning, it may be that the toxic properties of aggregated PrP^d^ or the loss of PrP^c^ function alter the axon–oligodendrocyte interaction. We observed PrP^d^ aggregates in oligodendrocytes in white matter, but not in cortical satellite oligodendrocytes. White matter myelin is traditionally considered to ensheath the axons of pyramidal neurons. Therefore, PrP^d^ aggregates transported along the axon might be taken-up by oligodendrocytes, a mechanism that has been suggested for oligodendrocyte alpha-synuclein accumulation in multiple system atrophy [24]. The axonal transport of PrP^d^ is also known to occur in the VV2 subtype where PrP^d^ accumulation can be identified along white matter axons and in perivascular areas [7,25,26]. Whether the MM2C patchy perivacuolar pattern affects a particular neuronal subtype that interacts more closely with oligodendrocytes, or whether in the M2C strain-specific PrP^d^ molecules transported from these cortical deposits in axons are more prone to be taken up by oligodendroglia in the white matter, is not clear and deserves further investigation.

In the late 1990s, El Hachimi et al. [27] identified PrP^d^ deposits in the white matter along myelin sheaths and in oligodendrocytes in four CJD cases (unspecified subtypes). Ultrastructural studies revealed the presence of dense osmiophilic, amorphous, partly fibrillar material associated with the lysosomes of oligodendrocytes. Moreover, Andres Benito et al. [28] reported an altered gene expression profile specific to astrocytes, oligodendrocytes and myelin in the frontal cortex of sCJD (7 MM1, 10 VV2), supporting the notion that molecular deficits linked to energy metabolism and solute transport in astrocytes and oligodendrocytes, in addition to neurons, may be relevant in the pathogenesis of cortical lesions in CJD. The authors also made similar observations in a murine CJD model [29].

In animals, a ramified astroglial PrP pattern is described for BSE and scrapie [20,21,22,23,24,25,26,27,28,29,30,31,32]. Particularly, in experimental TSE, the H-type BSE cases have been reported to show widespread glial labelling throughout the white matter of the spinal cord and the cerebellum [33]. Whether the similarities with some particular human disease forms with rare phenotypes might indicate some environmental influence on the disease phenotype remains unclear.

## 5. Conclusions

Oligodendroglial PrP^d^ pathology may be detected in the white matter of sCJD, particularly in those subtypes with abundant patchy-perivacuolar PrP type 2 aggregates (M2C “strain”), which are usually, but not necessarily, associated with longer disease duration.

## Figures and Tables

**Table 1:** Demographic and neuropathological characteristics of patients included in the study.

Case no.	Sex	Age at Death (years)	Disease Duration (months)	Histotype	Oligodendroglial PrP^d^ White Matter	Main PrP^d^ Pattern Frontal	Main PrP^d^ Pattern Occipital	Intensity PrP^d^ Deposits
1	f	72	3	MM/MV1	no	diffuse synaptic	diffuse synaptic	extensive
2	m	71	3	MM/MV1	no	diffuse synaptic deep laminar	diffuse synaptic	mild frontal, extensive occipital
3	m	72	3	MM/MV1	isolated frontal (severe cortical degeneration)	diffuse synaptic	diffuse synaptic	extensive
4	m	73	5	MM/MV1	no	Diffuse synaptic deep laminar	diffuse synaptic deep laminar	moderate
5	f	63	1.5	MM/MV1	no	diffuse synaptic	diffuse synaptic	extensive
6	m	62	6	MM/MV1	no	diffuse synaptic laminar	diffuse synaptic laminar	mild
7	f	57	3	MM/MV1	no	diffuse synaptic	diffuse synaptic	extensive
8	m	75	2	MM/MV1	no	diffuse synaptic	diffuse synaptic	mild
9	m	72	2	MM/MV1	no	diffuse synaptic	diffuse synaptic	moderate frontal, extensive occipital
10	m	72	2	MM/MV1	no	diffuse synaptic	diffuse synaptic	moderate
11	f	74	6	MM/MV1	no	diffuse synaptic	diffuse synaptic	extensive
12	f	67	2	MM/MV1	no	diffuse synaptic	diffuse synaptic	moderate
13	m	63	1	MM/MV1	no	diffuse synaptic	diffuse synaptic	moderate
14	f	67	3	MM/MV1	no	diffuse synaptic	diffuse synaptic	moderate
15	m	59	3.5	MM/MV1	no	diffuse synaptic	diffuse synaptic	moderate frontal, extensive occipital
16	m	56	2	MM/MV1	no	diffuse synaptic	diffuse synaptic	moderate
17	f	65	2	MM/MV1	no	diffuse synaptic	diffuse synaptic	extensive
18	f	54	2	MM/MV1	no	diffuse synaptic	diffuse synaptic	moderate, extensive occipital
19	m	77	2	MM/MV1	no	diffuse synaptic	diffuse synaptic	extensive frontal, mild occipital
20	f	65	1.5	MM/MV1	no	diffuse synaptic	diffuse synaptic	moderate
1	f	66	5	VV2	no	deep perineuronal	deep perineuronal	moderate
2	m	62	3	VV2	no	deep perineuronal	deep perineuronal	moderate
3	m	62	4	VV2	no	deep perineuronal	deep perineuronal	extensive
4	f	69	6	VV2	no	deep perineuronal	deep perineuronal + plaque-like	extensive
5	f	74	3	VV2	no	deep perineuronal + plaque-like	deep perineuronal + plaque-like	moderate
6	m	81	2	VV2	no	deep perineuronal + plaque-like	deep perineuronal	mild
7	m	74	3	VV2	no	deep perineuronal + plaque-like	deep perineuronal + plaque-like	extensive frontal, mild occipital
8	m	78	4	VV2	no	deep perineuronal	deep perineuronal	extensive frontal, moderate occipital
9	f	75	5	VV2	no	deep perineuronal	deep perineuronal	moderate frontal, mild occipital
10	f	80	3	VV2	no	deep perineuronal	deep perineuronal	moderate
1	m	74	7	MM/MV1+2C	yes, isol	diffuse synaptic + focal patchy	diffuse synaptic + patchy	moderate-extensive
2	m	65	17	MM/MV1+2C	no	diffuse synaptic	diffuse synaptic + focal patchy	extensive synaptic frontal, mild synaptic occipital
3	f	77	2	MM/MV1+2C	no	diffuse synaptic	diffuse synaptic + focal patchy	moderate
4	m	59	3	MM/MV1+2C	no	diffuse synaptic + focal patchy	diffuse synaptic + focal patchy	mild synaptic frontal, moderate synaptic occipital
5	m	81	2	MM/MV1+2C	no	diffuse synaptic	diffuse synaptic + focal patchy	mild synaptic
6	m	55	9	MM/MV1+2C	no	diffuse synaptic + patchy	diffuse synaptic	moderate frontal, extensive synaptic occipital
7	f	68	1	MM/MV1+2C	no	diffuse synaptic + focal patchy	diffuse synaptic + focal patchy	extensive synaptic
8	f	51	5	MM/MV1+2C	yes, few frontal	patchy perivacuolar	diffuse synaptic + focal patchy	extensive patchy frontal, extensive synaptic occipital
9	f	79	5	MM/MV1+2C	yes, few frontal	patchy perivacuolar	diffuse synaptic + focal patchy	extensive patchy frontal, extensive synaptic occipital
10	m	74	2	MM/MV1+2C	no	diffuse synaptic + focal patchy	diffuse synaptic + focal patchy	moderate synaptic
11	m	70	2	MM/MV1+2C	no	diffuse synaptic	diffuse synaptic + focal patchy	extensive synaptic frontal, moderate synaptic occipital
12	m	62	5	MM/MV1+2C	no	diffuse synaptic	diffuse synaptic + focal patchy	extensive
13	m	62	2	MM/MV1+2C	no	diffuse synaptic	diffuse synaptic	moderate
14	f	55	2	MM/MV1+2C	yes, few occipital	diffuse synaptic + patchy	diffuse synaptic + patchy	moderate frontal, extensive patchy occipital
15	f	96	n.a.	MM/MV1+2C	no	diffuse synaptic + focal patchy	diffuse synaptic + patchy	moderate-extensive
1	m	66	3	MV2K+C	no	mild	mild	mild
2	m	62	7	MV2K+C	yes	patchy perivacuolar	patchy perivacuolar	extensive
3	f	62	13	MV2K+C	no	patchy perivacuolar	patchy perivacuolar	moderate
4	m	78	8	MV2K+C	yes, few occipital	patchy perivacuolar	patchy perivacuolar	extensive
5	f	77	12	MV2K+C	yes, few occipital	patchy perivacuolar	patchy perivacuolar	extensive
6	m	70	18	MV2K+C	yes, isolated	patchy perivacuolar	patchy perivacuolar	moderate
7 *	m	63	31	MV2K+C	yes	patchy perivacuolar	patchy perivacuolar	extensive
8	m	57	2	MV2K+C	no	deep perineuronal	patchy perivacuolar + synaptic	moderate frontal, extensive occipital
9	f	73	9	MV2K+C	yes, few	moderate deep laminar + extensive patchy perivacuolar	moderate deep laminar + extensive patchy perivacuolar	moderate-extensive
10	m	57	57	MV2K+C	yes, isolated	moderate deep laminar + focal patchy perivacuolar	moderate deep laminar + extensive patchy perivacuolar	moderate frontal, extensive occipital
1	f	79	5	MM2C + 1	yes	patchy perivacuolar	patchy perivacuolar	extensive
2	f	52	58	MM2C	yes, extensive	patchy perivacuolar	patchy perivacuolar	extensive
3	f	80	2	MM2C	yes, few	patchy perivacuolar	patchy perivacuolar	moderate frontal, extensive occipital
4	m	78	22	MMC	yes	patchy perivacuolar	patchy perivacuolar	extensive
5	f	60	11	MM2C + 1	yes	patchy perivacuolar	patchy perivacuolar + synaptic	extensive
6	f	76	12	MM2C	yes, few	patchy perivacuolar	patchy perivacuolar	moderate
7	f	77	3	MM2C	no	patchy perivacuolar	patchy perivacuolar	moderate-extensive
8	f	64	12	MM2C	yes	patchy perivacuolar	patchy perivacuolar	moderate frontal, extensive occipital
9	f	60	2	MM2C + 1	yes	patchy perivacuolar	patchy perivacuolar + synaptic	extensive frontal, moderate occipital
10	f	51	11	MM2C	yes	patchy perivacuolar	patchy perivacuolar	extensive

* Patient in Figure 1M–O.

## Data Availability

The data presented in this study are available on request from the corresponding author. Some patients’ data are not publicly available due to privacy reasons.
